# *Camellia nitidissima* Flower Extract Alleviates Dermal Papilla Cell Dysfunction by Regulating 11β-HSD1 and the TGF-β2/Smad and Wnt/β-Catenin Pathways

**DOI:** 10.3390/cimb48090954

**Published:** 2026-09-18

**Authors:** Meng Zhang, Zhenyu Qin, Timson Chen, Zhizhen Li, Ya Chen, Ling Ma, Guangli Wang, Jing Wang

**Affiliations:** 1 Key Laboratory of Synthetic and Biological Colloids, Ministry of Education, School of Chemical & Material Engineering, Jiangnan University, Wuxi 214122, China; 6240606040@stu.jiangnan.edu.cn (M.Z.); 6240610035@stu.jiangnan.edu.cn (Z.Q.); 2 Adolph Innovation Laboratory, Guangzhou AOGU Cosmetics Co., Ltd., Guangzhou 510520, China; Timson_chen2020@126.com (T.C.); lizhizhen@adolph.cn (Z.L.); chenya@haservey.com (Y.C.); maling@adolph.cn (L.M.)

**Keywords:** *Camellia nitidissima* flower extract, HPA axis, 11β-HSD1, Wnt/β-catenin pathway, TGF-β2/Smad pathway

## Abstract

Psychological stress activates the local hypothalamic–pituitary–adrenal (HPA)-like axis in hair follicles, leading to cortisol overproduction and dysfunction of dermal papilla cells (DPCs). However, effective natural ingredients targeting this mechanism remain scarce. To investigate whether *Camellia nitidissima* flower extract (CNF) alleviates stress-induced DPC dysfunction by inhibiting 11β-Hydroxysteroid dehydrogenase type 1(11β-HSD1) and modulating the downstream signaling pathways. The phytochemical composition of CNF was characterized using HPLC-DAD. An in vitro stress-induced injury model was established in human DPCs using corticotropin-releasing factor (CRF). Cortisol levels as well as the secretion levels of hair growth factors were measured by ELISA. The expression of 11β-HSD1 was assessed via immunofluorescence. Oxidative stress was evaluated by reactive oxygen species (ROS) fluorescence and superoxide dismutase (SOD) activity. Apoptosis was analyzed by flow cytometry (Annexin V-FITC/PI). Protein expression of the TGF-β2/Smad pathway and mitochondrial apoptosis-related proteins was detected by western blotting. Cell proliferation was assessed by Ki67 immunofluorescence and cell cycle analysis. The mRNA expression of Wnt/β-catenin pathway components was quantified by Quantitative real-time reverse transcription polymerase chain reaction (qRT-PCR). Ten bioactive constituents, predominantly the polyphenolic compounds rutin and quercetin-7-O-β-D-glucoside, were identified in CNF. Mechanistically, CNF disrupted the local “stress-cortisol” amplification loop by first reducing CRF-induced cortisol secretion and downregulating 11β-HSD1 expression in DPCs. This upstream intervention subsequently attenuated oxidative stress and inhibited CRF-induced apoptosis via suppression of the TGF-β2/Smad2/3 pathway, while concurrently restoring proliferative capacity through reactivation of the Wnt/β-catenin signaling axis. These coordinated molecular events ultimately reinstated the secretion of key hair growth factors, including Alkaline phosphatase (ALP), Vascular endothelial growth factor (VEGF), Hepatocyte growth factor (HGF), Epidermal growth factor (EGF), and insulin-like growth factor 1 (IGF-1), thereby mitigating DPC dysfunction.

## 1. Introduction

The scalp provides the physiological bedrock for hair growth, and its homeostatic balance is a primary determinant of hair quality, which in turn serves as a conspicuous biomarker of both physical and mental health [1]. In modern society, the rising prevalence of hair disorders—including thinning, alopecia [2,3], and premature graying—has become a pressing concern across all age groups, severely undermining individuals’ self-esteem and social well-being [4,5].

As a complex mini-organ that sustains cyclical hair regeneration, the hair follicle (HF) relies critically on the functional integrity of its components. At the base of the follicle, dermal papilla cells (DPCs) act as the central signaling hub for hair growth regulation [6]. Through the secretion of diverse growth factors and cytokines, DPCs precisely orchestrate the proliferation and differentiation of follicular epithelial cells via paracrine signaling [7,8,9,10]. Notably, recent studies have identified a localized hypothalamic–pituitary–adrenal (HPA)-like axis within DPCs. Psychological stress elevates local corticotropin-releasing factor (CRF) levels, triggering downstream adrenocorticotropic hormone (ACTH) release [11,12,13]. Crucially, the local amplification of glucocorticoid signaling within this peripheral HPA-like axis depends on the enzyme 11β-hydroxysteroid dehydrogenase type 1 (11β-HSD1), which converts inactive cortisone into active cortisol, thereby establishing a self-perpetuating “stress-cortisol amplification loop” [14,15]. Chronic activation of this loop induces oxidative injury, cellular senescence, and functional decline in DPCs [16,17], ultimately driving follicular miniaturization and accelerated hair loss [17,18]. Despite its pathological relevance, research on cosmetic actives capable of modulating 11β-HSD1 via the HPA axis to counteract stress-induced scalp impairment remains remarkably limited [19].

*Camellia nitidissima* is an evergreen shrub or small tree characterized by its unique golden-yellow petals within the *Camellia* genus, earning it the moniker “Queen of the Camellia Family”. Approximately 90% of the wild *C. nitidissima* population is strictly endemic to the Lanshan branch of the Shiwandashan Mountains in Fangchenggang, Guangxi, China, making it an extraordinarily rare and precious botanical resource widely revered as the “Giant Panda of the Plant Kingdom”. The flowers of *C. nitidissima* are abundant in diverse bioactive phytochemicals-primarily comprising polyphenols, flavonoids, saponins, and polysaccharides, as well as essential vitamins and minerals-exhibiting immense potential for dermatological and personal care applications [20,21,22]. To date, sub stantial evidence has validated that *Camellia nitidissima* flowers possess robust antioxidant capacity, establishing them as an excellent naturally derived free-radical scavenger [23,24]. Unfortunately, the versatile application potential of *Camellia nitidissima* remains largely unexplored.

In this study, we aimed to investigate whether CNF exerts protective effects against CRF-induced DPC dysfunction and to elucidate the underlying molecular mechanisms. The present study demonstrates that *Camellia nitidissima* flower extract (CNF) effectively abolishes the local “stress-cortisol” amplification loop in human DPCs by specifically downregulating the expression and inhibiting the enzymatic activity of 11β-HSD1. The major novelty of this work lies in its pioneering focus on the follicle–resident HPA axis as a neuroendocrine metabolic target, systematically elucidating the profound molecular mechanisms through which CNF synergistically modulates the TGF-β2/Smad and Wnt/β-catenin pathways to restore the hair-inductive phenotype of DPCs. Consequently, this research not only broadens the translational trajectory of China’s endemic and rare botanical resources into anti-impairment scalp care but also provides an innovative, naturally derived solution and a robust cell-biological foundation for the intervention of contemporary psychological stress-induced alopecia.

## 2. Materials and Methods

### 2.1. Extraction, Purification and Phytochemical Characterization of CNF

#### 2.1.1. Extraction of CNF

*Camellia nitidissima* flowers were procured from Dongxing Zhengda Trading Co., Ltd. (Dongxing, China), a licensed supplier with legal collection authorization. The species was authenticated by the supplier and subsequently confirmed in our laboratory. A voucher specimen has been deposited at the Guangxi Museum of Natural Resources (Nanning, China). Fresh flowers were freeze-dried and mechanically pulverized into a fine powder. The powdered material was extracted with 55% ethanol at a solid-to-liquid ratio of 1:20 (*w*/*v*) in a 70 °C water bath for 3 h, and the extraction was repeated twice. The combined extracts were filtered through a 0.22 μm membrane to remove insoluble residues. The filtrate was concentrated under reduced pressure using a rotary evaporator to remove ethanol, yielding a viscous aqueous concentrate. The concentrate was further filtered and then spray-dried to obtain the final *Camellia nitidissima* flower extract (CNF) powder, which was stored in a desiccator at room temperature in the dark until further use.

#### 2.1.2. Phytochemical Characterization of CNF

The phytochemical profile of CNF was characterized using high-performance liquid chromatography coupled with a diode-array detector (HPLC-DAD). Chromatographic separation was performed on a Phenomenex C18 column (5 μm, 4.6 × 250 mm) via gradient elution with 0.1% aqueous formic acid (mobile phase A) and acetonitrile (mobile phase B) at a constant flow rate of 0.6 mL/min over 90 min. UV detection was monitored at wavelengths of 200, 254, and 350 nm. Major constituents were tentatively identified by comparing their retention times and UV absorption spectra with those of authentic reference standards.

### 2.2. Cell Culture

Human dermal papilla cells (DPCs) were purchased from Zhejiang Meisen Cell Technology Co., Ltd. (Jinhua, China) and cultured in DF12 medium supplemented with 10% fetal bovine serum (Gibco, Carlsbad, CA, USA), 1.0 × 10^5^ U/L of penicillin, and 100 mg/L of streptomycin. The culture conditions were set at 37 °C, 5% CO_2_, and saturated humidity. For subculture, cells were digested with 0.25% EDTA-containing trypsin solution (Gibco) and passaged in fresh complete medium described above.

### 2.3. Assay for Cell Viability

The 3-(4,5-dimethylthiazol-2-yl)-2,5-diphenyltetrazolium bromide (MTT) assay [25] was used to evaluate the cytotoxicity of CNF and CRF (Sigma-Aldrich CO., Saint Louis, MO, USA). Stock solutions of the samples were prepared as follows: 50 mg/mL CNF was obtained by dissolving 500 mg of CNF in 10 mL of DF12 medium; 1.0 mg/mL CRF was prepared by dissolving 2.0 mg of CRF in 2.0 mL of DF12 medium. The above stock solutions were serially diluted with high-glucose DF12 medium to obtain sample solutions with different mass concentrations.

DPCs in the logarithmic growth phase were digested, counted, and seeded in 96-well plates at a density of 1 × 10^5^ cells/mL with a cell suspension volume of 100 μL per well. After incubation at 37 °C in a 5% CO_2_ incubator (Bosun Medical Biological Instrument Co., Shanghai, China) for 24 h, 100 μL of sample solutions with different mass concentrations dissolved in DF12 medium were added to the experimental groups, and 100 μL of DF12 medium was added to the blank control group. After further incubation for 24 h, the cell supernatant was discarded, and the MTT (Sigma-Aldrich CO., Saint Louis, MO, USA) (100 μL, 0.5 mg/mL) was added to each well. After incubation in the incubator for 4 h, the supernatant was discarded, and 100 μL of DMSO (Sinopharm Group Chemical Reagents Co. Ltd., Beijing, China) was added to each well. The plates were shaken for 5 to 10 min in a microplate reader (Tecan Infinite 200Pro, Maennedorf, Switzerland), and the absorbance was measured at 490 nm. The cell viability was calculated according to the following Formula (1):

(1) $$\mathrm{Cell}\;\mathrm{viability}(\%)\;=\;\frac{A_{1}}{A_{2}}\;\times \;100\%$$

where A_1_ and A_2_ represent the average absorbance of the experimental group and the control group at 490 nm, respectively.

### 2.4. Measurement of Cortisol Secretion in CRF-Stimulated DPCs

To investigate the dose-dependent effect of CRF on cortisol production, DPCs in the logarithmic growth phase were harvested, counted, and seeded into 6-well plates at a density of 2 × 10^5^ cells/mL. After a 24-h incubation period, the culture medium was replaced with fresh medium containing varying concentrations of CRF (0.01, 0.1, and 1.0 μM), while the blank control group received only DF12 medium. Following an additional 24 h of treatment, the cell culture supernatants were collected. The cortisol concentration in each sample was subsequently quantified using a cortisol ELISA kit (Absin, Shanghai, China), strictly adhering to the manufacturer’s protocol, to establish the correlation between CRF stimulation and cortisol biosynthesis in DPCs.

### 2.5. Cell Treatment and Experimental Grouping Setting

For experimental treatments, DPCs were seeded into 6-well plates at a density of 2 × 10^5^ cells/mL and incubated for 24 h to allow adherence. The cells were then chronologically assigned into three major operational frameworks: (1) Blank control group, received fresh DF12 medium alone; (2) Model group, exposed to 1.0 μM of CRF (Sigma-Aldrich, St. Louis, MO, USA); and (3) Experimental groups, co-treated with 1.0 μM of CRF and specified gradient concentrations of CNF (25 or 50 μg/mL). All the groups were incubated for an additional 24 h before undergoing subsequent biomolecular extractions and assays.

### 2.6. Attenuation of Oxidative Stress by CNF

Following the intervention protocols described in Section 2.5. The DCFH-DA probe [26] (Beyotime Biotechnology, Shanghai, China) was diluted with serum-free medium at a ratio of 1:1000 to a final concentration of 10 μM. The DF12 medium was removed, and 1.0 mL of diluted DCFH-DA working solution was added to each well, followed by incubation at 37 °C in the dark for 20 min. The cells were washed three times with PBS, and images were captured using an inverted fluorescence microscope (Olympus IX73, Olympus Corporation, Tokyo, Japan).

DPCs were inoculated into 6-well plates (2 × 10^5^ cells/mL, 2.0 mL per well) and cultured for 24 h. The cells were then exposed to a mixture of CRF and CNF extract for an additional 24 h. Subsequently, the cells were harvested and lysed to collect the cellular extract. Intracellular superoxide dismutase (SOD) activity was evaluated using the corresponding ELISA kit [27] in strict accordance with the manufacturer’s protocol (Absin, Shanghai, China).

### 2.7. Immunofluorescence Staining

DPCs in the logarithmic growth phase were digested with trypsin, and the cell suspension density was adjusted to 5 × 10^4^ cells/mL, and 1.0 mL of the cell suspension was added to each confocal dish. After 24 h of culture, 1.0 mL of DF12 medium was added to the blank group, 1.0 mL of 1.0 μM CRF was added to the model group, and 1.0 mL of mixed solution containing 1.0 μM CRF and 50 μg/mL CNF was added to the experimental group. After 24 h of culture, the cells were washed three times with PBS buffer and fixed with 4% paraformaldehyde at room temperature for 15 min. After another three washes with PBS, blocking buffer was added for blocking at room temperature for 1 h. The blocking solution was removed, and specific primary antibodies [28] against 11β-HSD1, marker of proliferation Ki-67 (Ki67), and growth arrest-specific protein 6 (GAS6) were added and incubated at 4 °C overnight. The next day, after washing with PBS, secondary antibodies were added and incubated at room temperature in the dark for 2 h. After staining, the cell nuclei were labeled with DAPI staining [29,30] solution for 10 min, and images were captured using a laser scanning confocal microscope (TCS SP8, Leica Microsystems, Wetzlar, Germany).

### 2.8. Detection of Cell Apoptosis by Annexin V-FITC/PI Double Staining

Following the intervention protocols described in Section 2.5, cells were harvested by centrifugation (1500 rpm, 5 min) and washed twice with ice-cold PBS. For apoptosis detection, cells were resuspended in 200 μL of Binding Buffer, incubated with 5.0 μL of Annexin V-FITC at 2–8 °C for 15 min in the dark, and stained with 10 μL of propidium iodide (PI) [31] for 5 min. For cell cycle analysis, washed cells were fixed in 70% pre-cooled ethanol at 4 °C for 12 h; after removing the fixative via PBS washing, cell pellets were resuspended in PI staining [1] solution and incubated at 37 °C in the dark for 30 min. Finally, apoptosis rates and cell cycle distributions (including the cell proliferation index) [32] were acquired on a flow cytometer (BD FACSAria III, BD Biosciences, San Jose, CA, USA) and analyzed using FlowJo software (BD FACSDiva 8.0.2).

### 2.9. Measurement of Secreted Factors and Cellular Markers in DPCs

Following the cell grouping and treatment protocols detailed in Section 2.5, culture supernatants were harvested for subsequent analysis. The concentrations of vascular endothelial growth factor (vegf), epidermal growth factor (egf), hepatocyte growth factor (hgf), insulin-like growth factor-1 (igf-1), and alkaline phosphatase (alp) in the culture medium were determined according to the instructions of the ELISA kits (Beyotime Biotechnology, Shanghai, China).

### 2.10. Detection of mRNA Expression by Quantitative Real-Time RT-PCR (qRT-PCR)

Following the cell intervention protocols described in Section 2.5, total RNA was extracted from DPCs using Trizol reagent, and qRT-PCR was performed using the BeyoFast™ SYBR Green One-Step RT-PCR Kit (Beyotime Biotechnology, Shanghai, China). With β-actin as the internal reference gene, the relative mRNA expression levels were calculated and normalized by the 2^−ΔCt^ method. The design and synthesis of primers for the target genes and the internal reference gene in this experiment were commissioned to Servicebio Technology Co., Ltd. (Wuhan, China). The primers used are shown in Table 1.

### 2.11. Western Blotting

Following the intervention protocols described in Section 2.5, the culture medium was aspirated, and total proteins were extracted using RIPA lysis buffer, and protein concentrations were determined via the BCA Protein Assay Kit. Equal amounts of protein were separated by SDS-PAGE and transferred onto 0.2 μm PVDF membranes. The membranes were blocked with 5% non-fat milk or 1% BSA for 2 h at room temperature, followed by overnight incubation at 4 °C with primary antibodies against TGF-β2, Smad 2, p-Smad 2, Smad 3, p-Smad 3, Caspase-3, Bcl-2, and Bax (all at 1:1000 dilution; Sanying Biotechnology, Wuhan, China). After washing with TBST, membranes were incubated with HRP-conjugated secondary antibodies (1:1000) for 1 h. Protein bands were visualized using an ECL kit (Beyotime Biotechnology) via the Chemi DOC XRS+ imaging system. β-Actin was used as the internal control for normalization.

### 2.12. Statistical Analysis

The data analysis was carried out using GraphPad, and the data were expressed in the form of mean ± standard deviation. All experiments were independently repeated at least three times (biological replicates, *n* ≥ 3). The between-groups comparison was assessed through analysis of variance (ANOVA) followed by Tukey’s test. A *p*-value less than 0.05 was considered to be statistically significant.

## 3. Results

### 3.1. Chemical Composition Analysis of CNF

**Figure 1:**
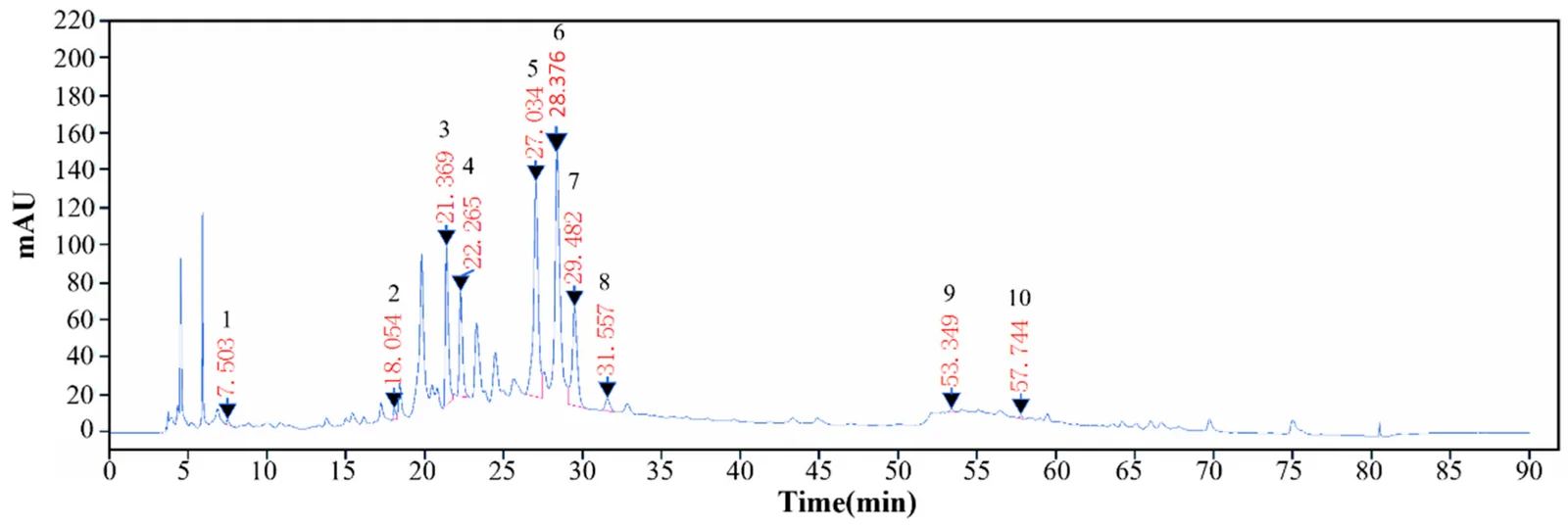
HPLC analysis for CNF.

The phytochemical profile of CNF was characterized via HPLC-DAD analysis (Figure 1). Although some high-polarity unlabeled peaks (putatively saccharides or organic acids) were present at the early elution phase, our analysis strategically focused on the polyphenolic and flavonoid fractions responsible for the core bioactivities. Based on comparison of retention times and UV spectra with authenticated reference standards, 10 characteristic bioactive constituents were successfully identified (Table 2). Rutin and quercetin-7-O-β-D-glucoside were identified as the predominant constituents, while several trace flavonoids were also detected. It is postulated that the robust cytoprotective efficacy of CNF against CRF-induced injury relies not solely on the high-abundance components, but rather on the holistic and synergistic molecular network formed by both major and trace polyphenolic flavonoids. This identification provides a chemical foundation for understanding the bioactive components of CNF.

### 3.2. Effects of CNF and CRF on the Viability of DPCs

**Figure 2:**
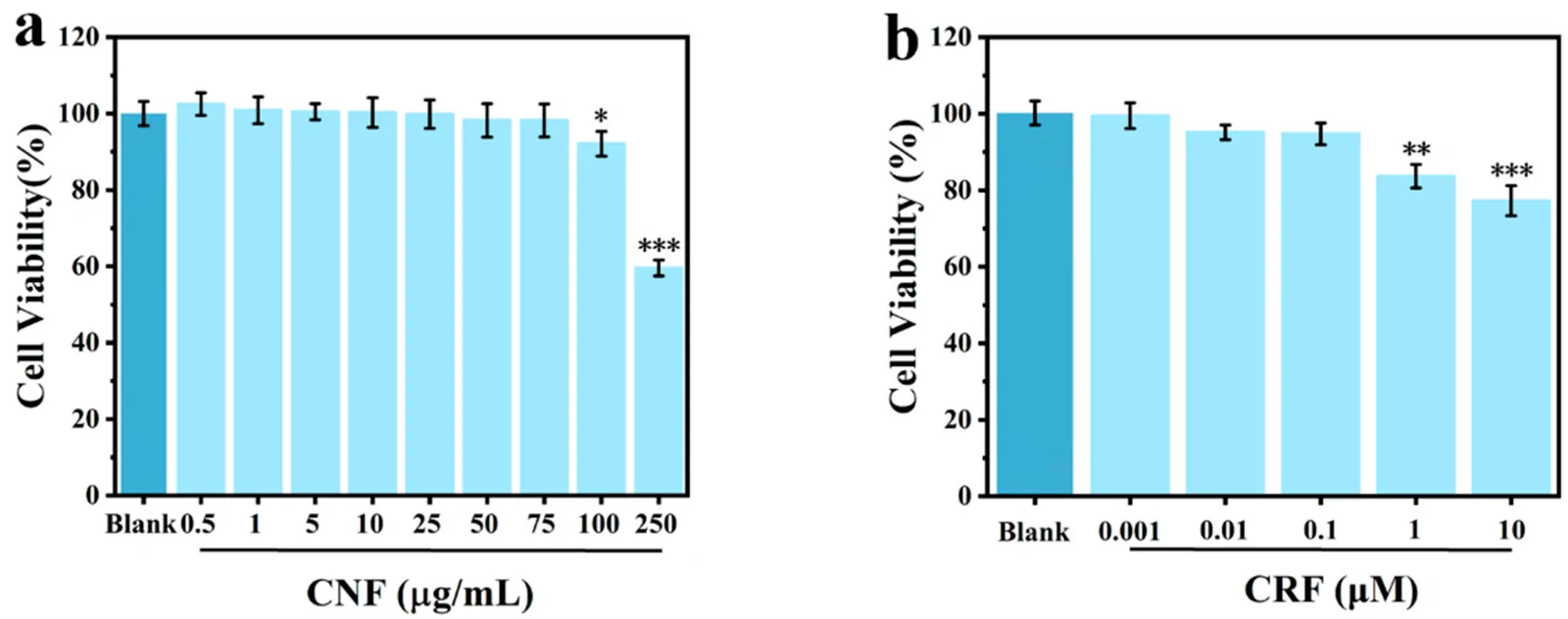
Effects of CNF and CRF on the viability of DPCs: (**a**) Cytotoxicity of CNF at different concentrations; (**b**) Cytotoxicity of CRF at different concentrations (*, **, and *** indicate *p* < 0.05, 0.01, and 0.001 vs. Blank group). All data are presented as mean ± SD from three independent biological replicates (*n* = 3).

First, the concentration-dependent cytotoxicity ranges of CNF and CRF on DPCs were detected by the MTT assay. The results shown in Figure 2 indicated that the viability of DPCs was higher than 80% at low concentration ranges (CNF ≤ 100 μg/mL; CRF ≤ 1.0 μM). Therefore, 25, 50, and 75 μg/mL of CNF, and 0.01, 0.1, and 1.0 μM of CRF were selected as the concentration ranges for subsequent experiments.

### 3.3. CNF Downregulates CRF-Induced 11β-HSD1 Expression and Cortisol Overproduction

**Figure 3:**
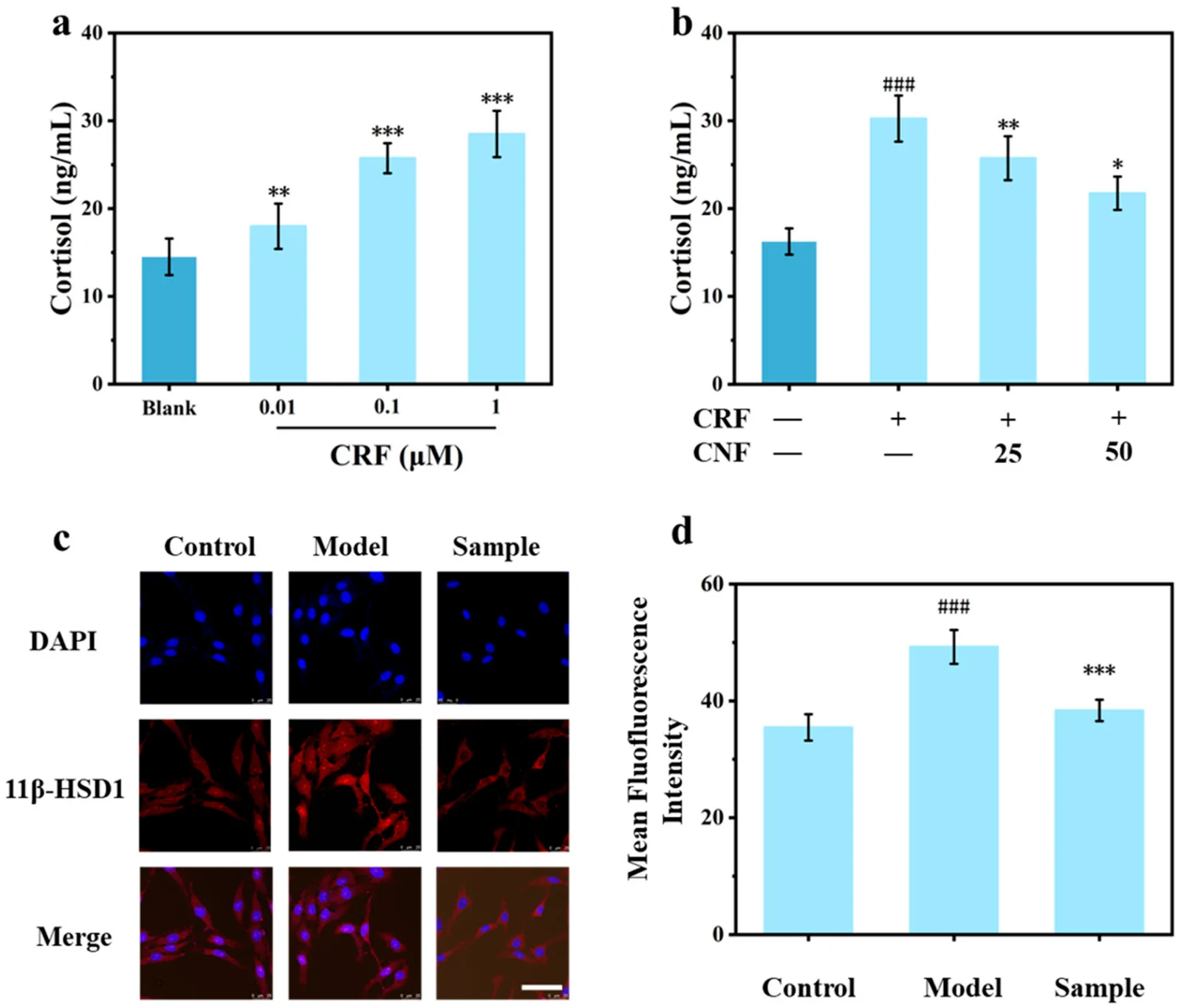
CNF downregulates CRF-induced 11β-HSD1 expression and cortisol overproduction. (**a**) Dose-dependent effects of CRF (0.01, 0.1, and 1.0 μM) stimulation on cortisol secretion in DPCs; (**b**) CNF (25,50 μg/mL) downregulates CRF-induced (1.0 μM) cortisol overproduction; (**c**) Immunofluorescence staining images of 11β-HSD1, Scale bars = 50 μm, All images are shown at the same scale bar; (**d**) Corresponding mean fluorescence intensity for 11β-HSD1 under different conditions: Control (untreated), Model (1.0 μM CRF), and CNF (1.0 μM CRF + 50 μg/mL CNF) (### indicated *p* < 0.001 vs. Control group; *, **, and *** indicated *p* < 0.05, 0.01, and 0.001 vs. Model group). All data are presented as mean ± SD from three independent biological replicates (*n* = 3).

To elucidate the dose–response relationship between CRF stimulation and cortisol production, DPCs were treated with varying concentrations of CRF, and cortisol levels in the culture supernatants were quantified via ELISA. As illustrated in Figure 3a, CRF treatment significantly promoted cortisol secretion in a dose-dependent manner, with increments of 24.14%, 77.58%, and 96.65% at 0.01, 0.1, and 1.0 μM CRF, respectively, compared to the untreated control, indicating that CRF effectively activates the local HPA axis in DPCs in a concentration-dependent manner. Given that 1.0 μM CRF elicited the most pronounced induction, this concentration was established as the standard for subsequent experimental modeling.

Furthermore, the inhibitory effect of CNF on CRF-induced cortisol biosynthesis. As shown in Figure 3b, the addition of 50 μg/mL of CNF resulted in a significant reduction in cortisol levels compared to the model group (1.0 μM of CRF), with a decrease of 28.10%, suggesting that CNF effectively suppresses CRF-induced cortisol overproduction in DPCs, likely through modulation of 11β-HSD1-dependent glucocorticoid metabolism. Collectively, these findings demonstrate that CNF effectively suppresses CRF-induced cortisol overproduction in DPCs. It is hypothesized that this regulatory effect may be mediated by the modulation of 11β-HSD1-dependent glucocorticoid metabolism, thereby contributing to the mitigation of cutaneous stress responses.

To further explore the mechanism by which CRF induces cortisol overproduction, whether CRF acts through the 11β-HSD1 mediated local HPA axis. The expression level of 11β-HSD1 was evaluated via immunofluorescence staining. Compared with the CRF-alone-treated group, co-treatment with 50 μg/mL of CNF resulted in a 22.1% reduction in 11β-HSD1 protein expression (Figure 3d, *** *p* < 0.001), confirming that CNF downregulates 11β-HSD1 expression at the protein level. These results demonstrated that CRF significantly upregulated 11β-HSD1 expression, whereas CNF reversed this upregulation. Collectively, these findings suggest that CNF suppresses CRF-induced cortisol biosynthesis, at least in part, by downregulating 11β-HSD1 expression in DPCs. By targeting this key regulatory node, CNF interrupts the stress signals mediated by the local HPA axis, thereby restoring the homeostasis of the hair growth microenvironment.

### 3.4. CNF Alleviates CRF-Induced Oxidative Stress

**Figure 4:**
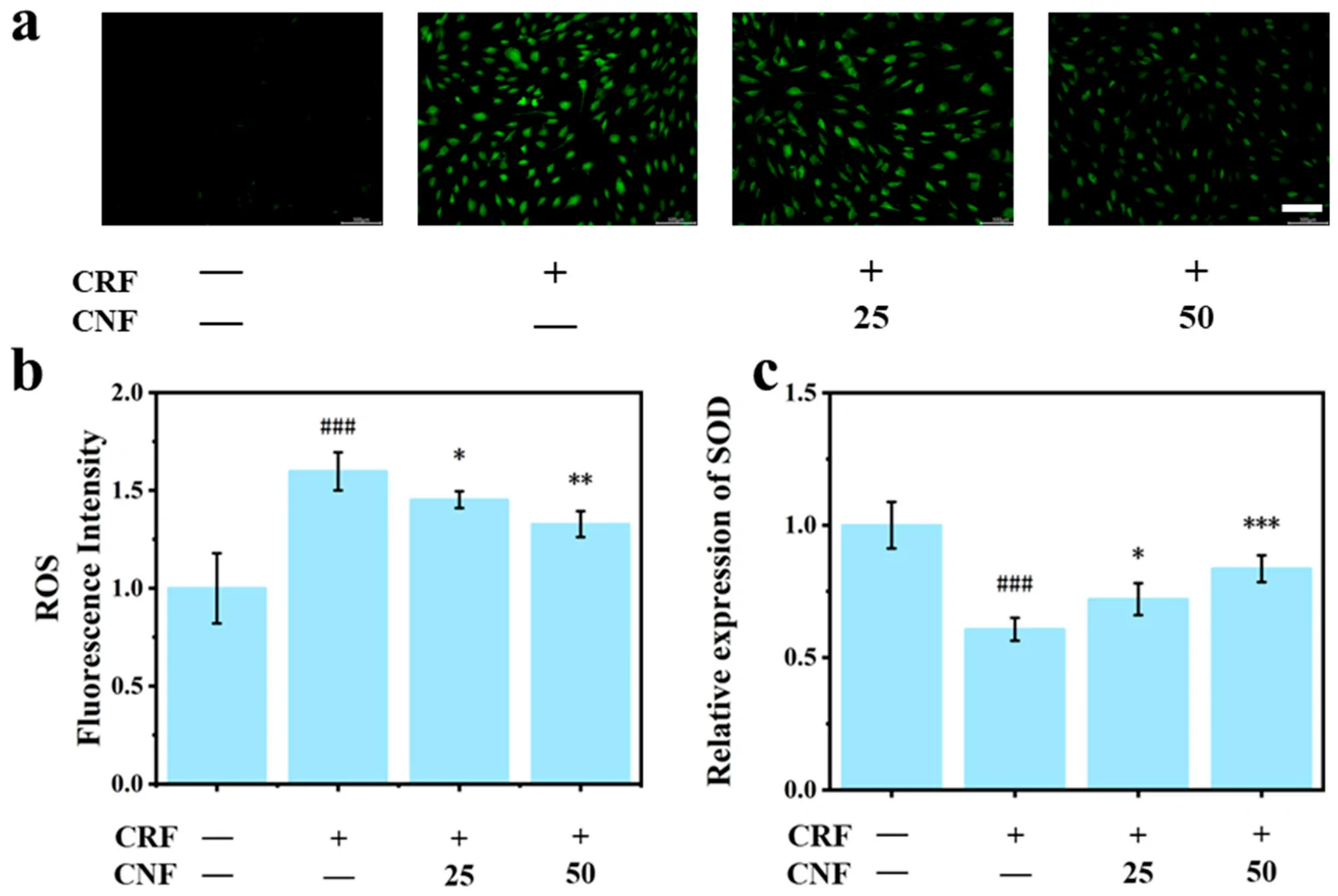
Effects of CNF on CRF-induced oxidative stress in DPCs. Cells were treated with 1.0 μM CRF and CNF at concentrations of 0, 25, or 50 μg/mL. (**a**) Representative fluorescence micrographs of intracellular ROS levels; (**b**) Relative fluorescence intensity of ROS, Scale bars = 500 μm, All images are shown at the same scale bar; (**c**) Relative expression of SOD (### indicated *p* < 0.001 vs. Control group; *, **, and *** indicated *p* < 0.05, 0.01, and 0.001 vs. Model group). All data are presented as mean ± SD from three independent biological replicates (*n* = 3).

Intracellular reactive oxygen species (ROS) levels were quantified using the DCFH-DA fluorometric assay. The results revealed that CRF stimulation triggered a profound accumulation of ROS within DPCs (Figure 4a). Conversely, intervention with CNF effectively abrogated this oxidative surge; specifically, treatment with 50 μg/mL CNF elicited a 17% reduction in ROS levels (Figure 4b, *p* < 0.01), underscoring the potent radical-scavenging capacity of the extract. Furthermore, as a hallmark of the endogenous antioxidant defense system, superoxide dismutase (SOD) activity was significantly suppressed following CRF stimulation. Remarkably, CNF treatment reversed this decline, with the 50 μg/mL dosage bolstering SOD activity by 36% (Figure 4c, *p* < 0.001). Collectively, these findings demonstrate that CNF confers robust protection against oxidative damage through a dual-action mechanism—the direct sequestration of CRF-induced ROS and the restoration of endogenous antioxidant enzymatic function—thereby alleviating cellular oxidative stress.

### 3.5. CNF Inhibits Cell Apoptosis by Modulating the TGF-β2/Smad Signaling Pathway

**Figure 5:**
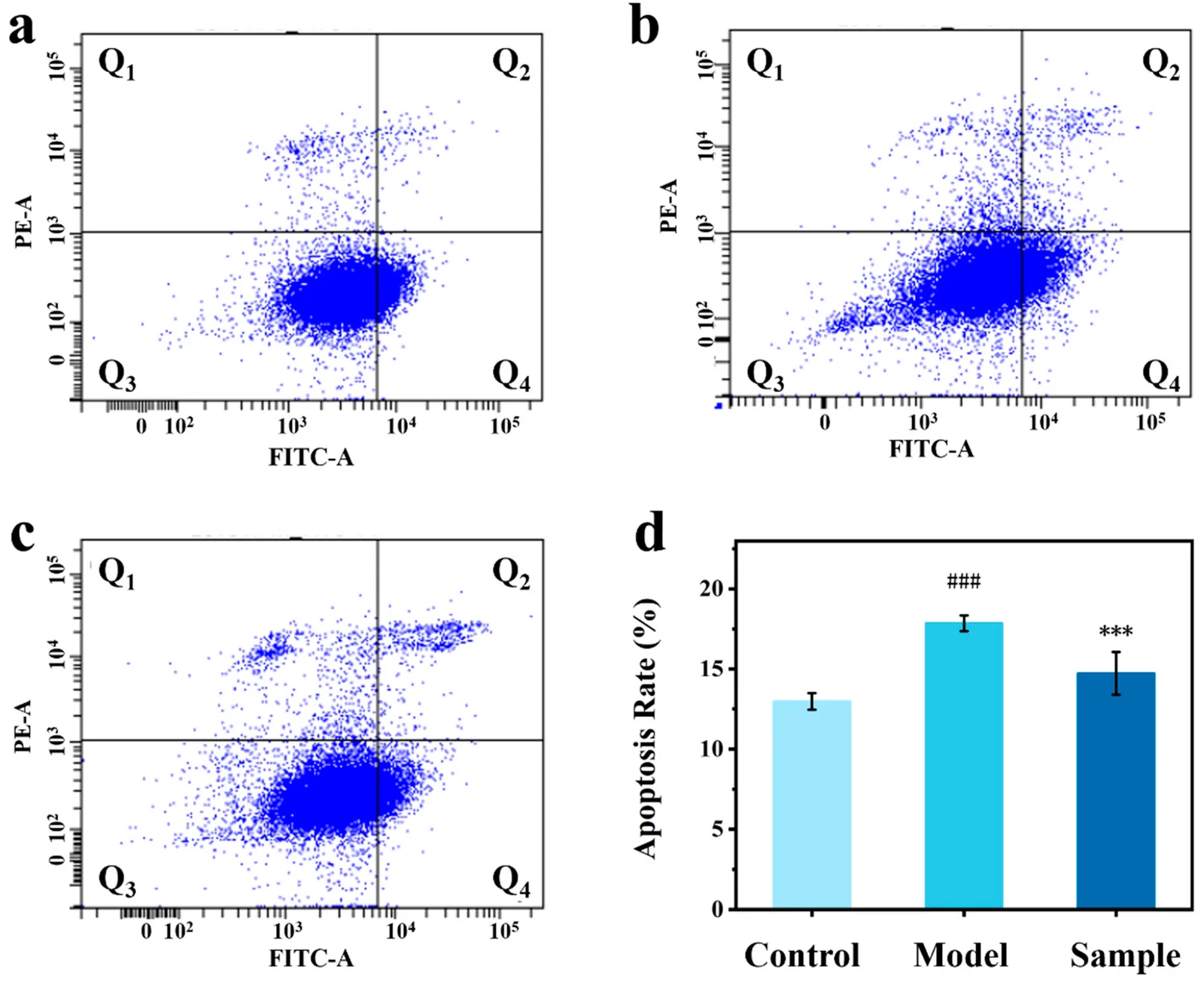
Assessment of DPC apoptosis by flow cytometry. (**a**–**c**) Representative flow cytometric plots of DPCs in (**a**) the control group, (**b**) the CRF-induced (1.0 μM) model group, (**c**) the group treated with CNF (50 μg/mL); (**d**) Quantitative analysis of apoptotic rates across the indicated groups (### indicated *p* < 0.001 vs. Control group; *** indicated *p* < 0.001 vs. Model group). All data are presented as mean ± SD from three independent biological replicates (*n* = 3).

Annexin V-FITC/PI staining and flow cytometric analysis confirmed that CRF (1.0 μM) significantly triggered apoptosis in DPCs, elevating the apoptotic rate by 37.3%. Conversely, treatment with 50 μg/mL CNF markedly attenuated this effect, reducing apoptosis by 17.6% (Figure 5d), indicating that CNF exerts a potent anti-apoptotic effect against CRF-induced programmed cell death in DPCs.

**Figure 6:**
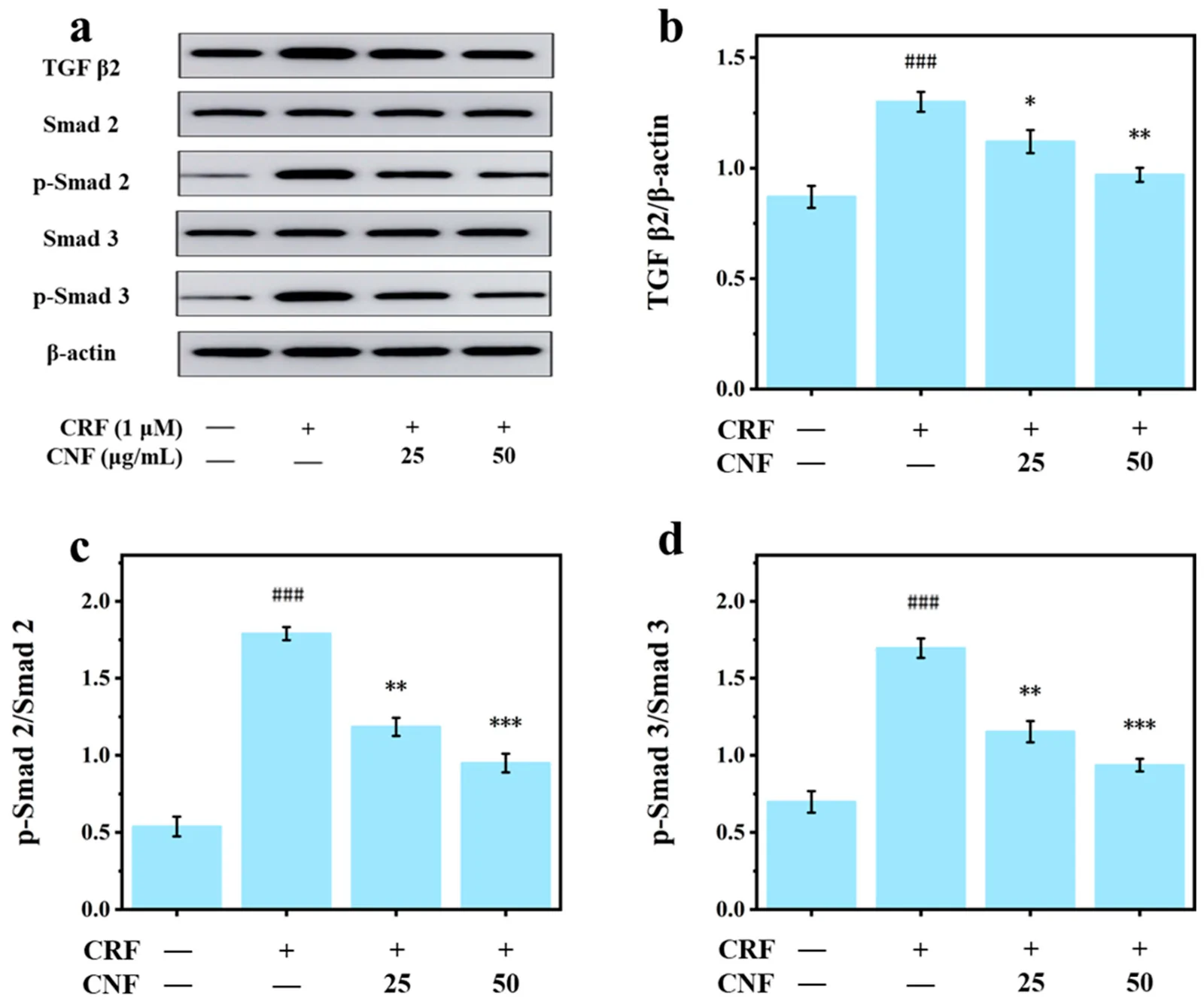
CNF attenuates CRF-induced activation of the TGF-β2/Smad signaling pathway. Cells were treated with 1.0 μM CRF and CNF at concentrations of 0, 25, or 50 μg/mL. (**a**) Western blot bands and corresponding band intensity for (**b**) TGF-β2/β-actin, (**c**) p-Smad 2/Smad 2 ratio, and (**d**) p-Smad 3/Smad 3 ratio (### indicated *p* < 0.001 vs. control group; *, **, and *** indicated *p* < 0.05, 0.01, and 0.001 vs. CRF group). All data are presented as mean ± SD from three independent biological replicates (*n* = 3).

To further elucidate the molecular mechanisms underlying CRF-induced apoptosis, the TGF-β2/Smad signaling pathway, a cascade intimately associated with the catagen phase of the hair follicle cycle, was assessed. Our analyses revealed that CRF treatment significantly upregulated protein expression levels of TGF-β2 in DPCs (Figure 6). Concomitantly, the phosphorylation of Smad 2/3, the principal downstream effectors of TGF-β2, was markedly enhanced without altering total Smad 2/3 protein levels, confirming that CRF specifically activates this signaling axis. Notably, administration of CNF effectively abrogated the CRF-induced upregulation of TGF-β2 and its downstream Smad 2/3 phosphorylation, restoring their expression and activation states to near-baseline control levels.

**Figure 7:**
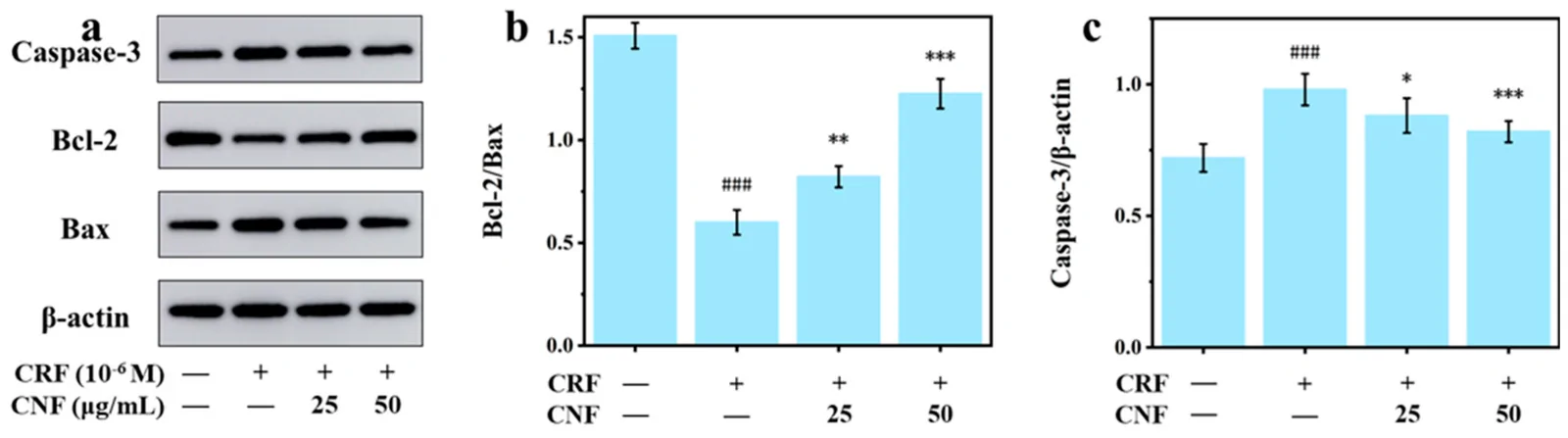
CNF suppresses the apoptotic pathway in CRF-treated DPCs. Cells were treated with 1.0 μM CRF and CNF at concentrations of 0, 25, or 50 μg/mL. (**a**) Western blot bands and corresponding band intensity for (**b**) Bcl-2/Bax ratio and (**c**) relative expression level of Caspase-3 (### indicated *p* < 0.001 vs. control group; *, **, and *** indicated *p* < 0.05, 0.01, and 0.001 vs. Model group). All data are presented as mean ± SD from three independent biological replicates (*n* = 3).

Given that sustained activation of the TGF-β2/Smad signaling axis frequently triggers the downstream mitochondrial apoptotic cascade, we next evaluated the expression of key regulatory proteins within this pathway. Our findings indicate that CRF exposure profoundly disrupts the intracellular pro-and anti-apoptotic equilibrium. Specifically, the upregulation of the pro-apoptotic protein Bax and the concurrent downregulation of the anti-apoptotic protein Bcl-2 resulted in a precipitous decline in the Bcl-2/Bax ratio-a critical molecular switch governing the initiation of mitochondrial apoptosis. This shift subsequently provoked robust activation of the downstream executioner protein, Caspase-3. Notably, the introduction of CNF significantly reversed this pro-apoptotic trajectory. By restoring Bcl-2 expression and suppressing Bax, CNF effectively rescued the Bcl-2/Bax ratio, thereby abrogating the downstream Caspase-3 activation cascade (Figure 7). Taken together, these results demonstrate that CNF confers protection against CRF-induced apoptosis by dampening the hyperactivation of the TGF-β2/Smad signaling pathway, which in turn modulates the mitochondrial apoptotic cascade to preserve cellular viability.

### 3.6. CNF Rescues CRF-Induced Proliferation Inhibition and Cell Cycle Arrest in DPCs via the Wnt/β-Catenin Signaling Pathway

**Figure 8:**
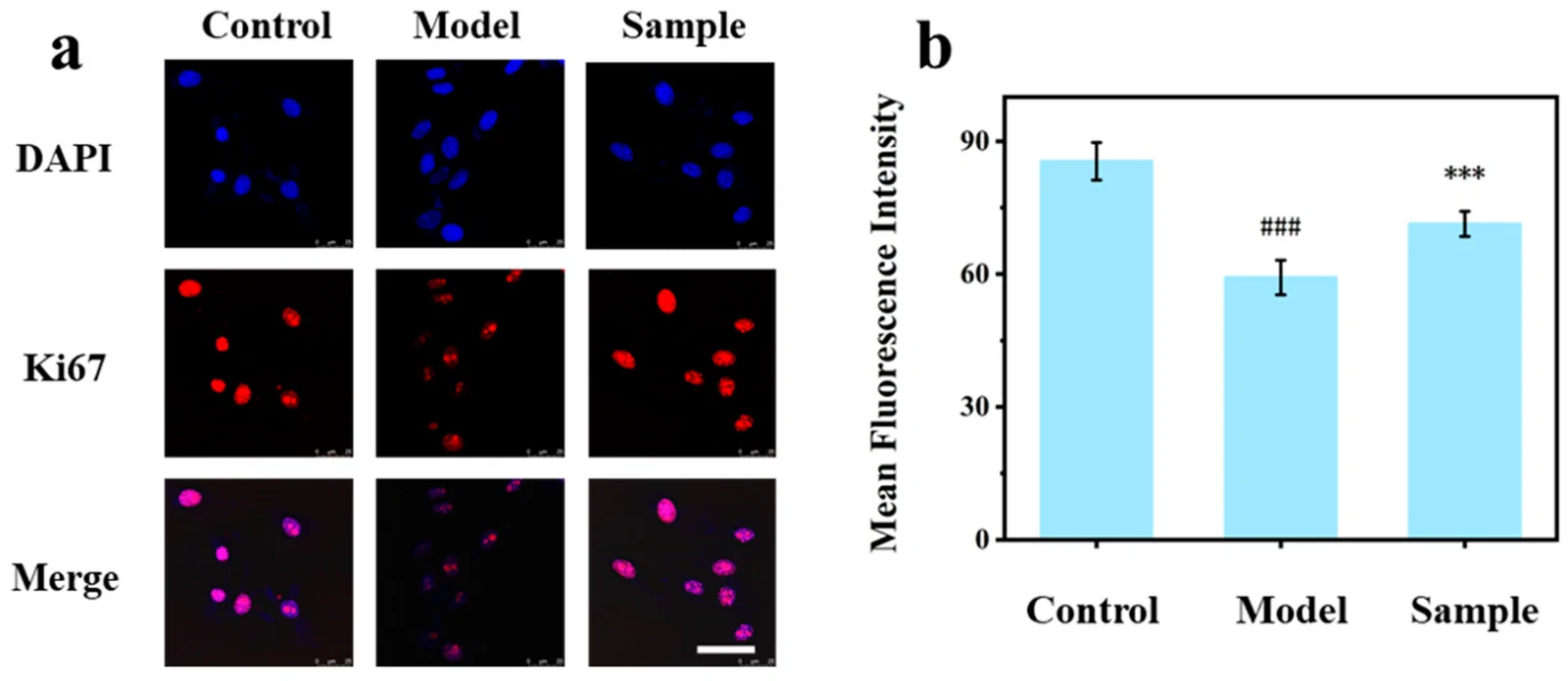
Effects of CNF on cell proliferation under different conditions: Control (untreated), Model (1.0 μM CRF), and CNF (1.0 μM CRF + 50 μg/mL CNF). (**a**) Immunofluorescence image of Ki67, Scale bars = 50 μm, All images are shown at the same scale bar; (**b**) Corresponding mean fluorescence intensity for Ki67 (### indicated *p* < 0.001 vs. Control group; *** indicated *p* < 0.001 vs. Model group). All data are presented as mean ± SD from three independent biological replicates (*n* = 3).

Immunofluorescence staining revealed that CNF effectively rescued the expression of the proliferation marker Ki67 (Figure 8), which was markedly suppressed by CRF. This result indicated that CNF restored the proliferative activity of DPCs at the single-cell level.

**Figure 9:**
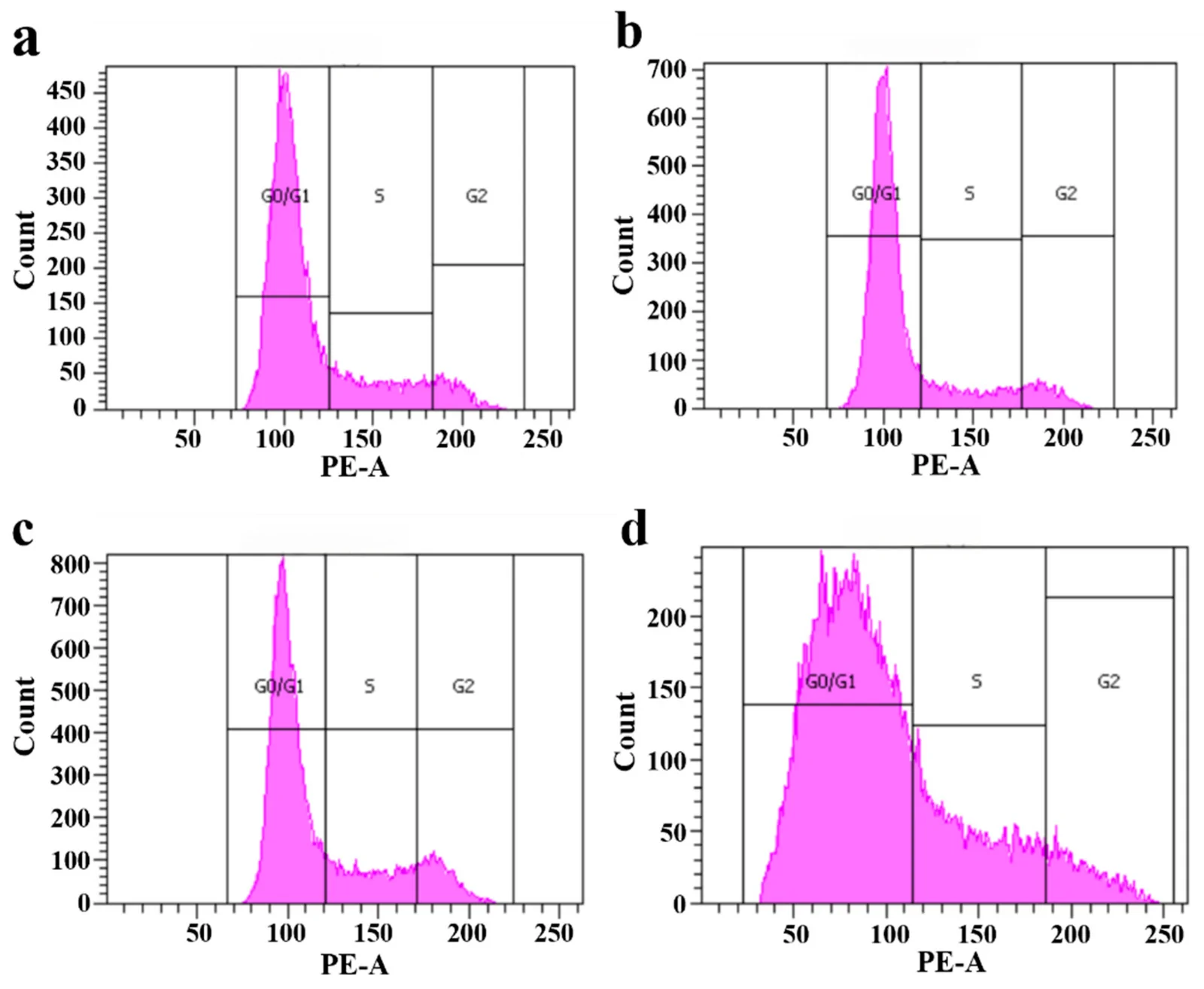
Cell cycle analysis via flow cytometry. (**a**) Blank control group; (**b**) CRF-induced model group (1.0 μM); (**c**) Experimental group treated with 1.0 μM CRF and 25 μg/mL CNF; (**d**) Experimental group treated with 1.0 μM CRF and 50 μg/mL CNF. All data are presented as mean ± SD from three independent biological replicates (*n* = 3).

To further elucidate this cytodynamic effect and determine whether the increased Ki67 expression was associated with altered cell cycle progression, propidium iodide (PI) staining coupled with flow cytometry was performed. As shown in Figure 9, CRF triggered a profound G0/G1 phase arrest, thereby impeding progression into the mitotic phases. Conversely, CNF treatment significantly abrogated this inhibitory effect, as evidenced by a robust shift of the cell population from the G0/G1 phase into the S and G2/M phases. These findings further confirmed that CNF promotes DPC proliferation by driving cell cycle transition from quiescence toward active DNA synthesis and mitosis.

**Figure 10:**
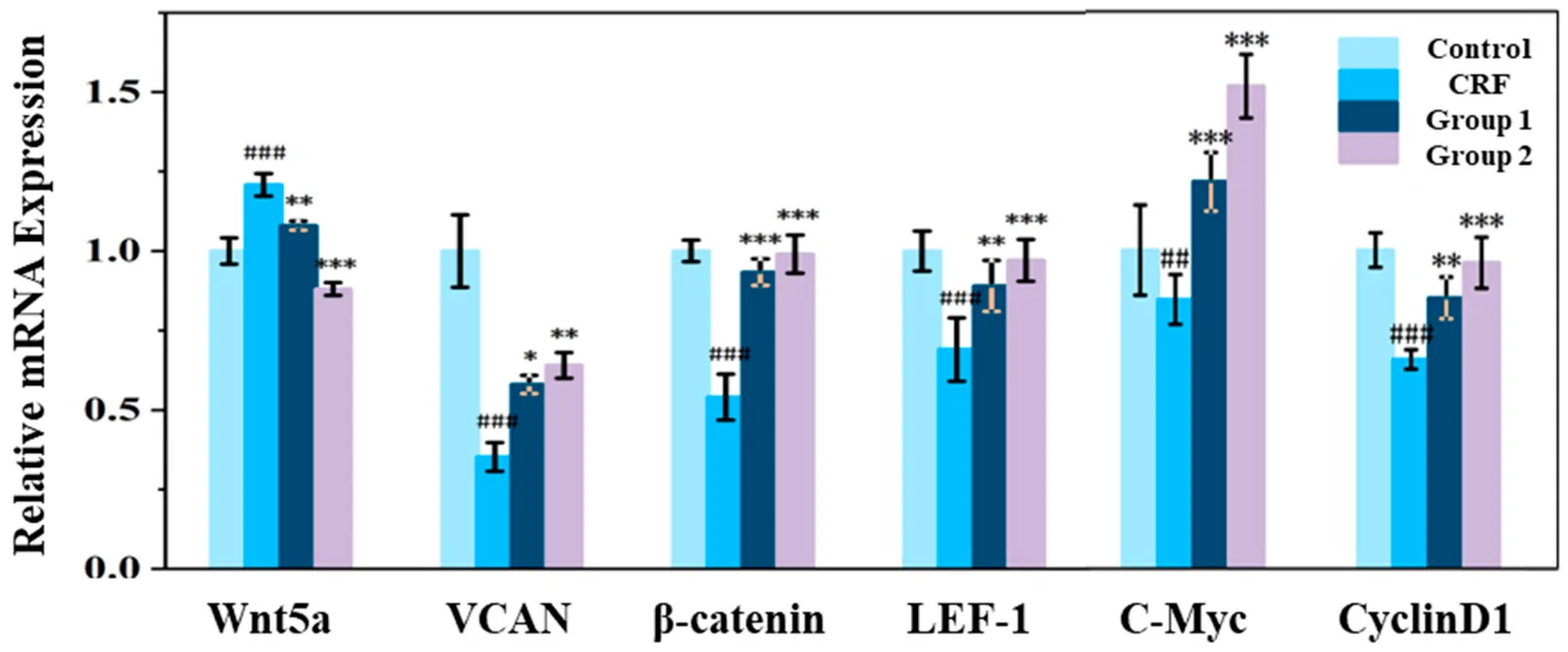
CNF restores the expression of Wnt/β-catenin signaling effectors (CRF-induced model group (1.0 μM), Group 1 treated with 1.0 μM CRF and 25 μg/mL CNF, Group 2 treated with 1.0 μM CRF and 50 μg/mL CNF) (##, ### indicated *p* < 0.01 and 0.001 vs. control group; *, **, and *** indicated *p* < 0.05, 0.01, and 0.001 vs. CRF group). All data are presented as mean ± SD from three independent biological replicates (*n* = 3).

To elucidate the underlying molecular mechanisms, the impact of CNF on the core regulatory pathways governing hair growth was assessed. qPCR analysis revealed that CRF significantly downregulated the expression of Wnt5a, a pivotal Wnt ligand, thereby arresting the Wnt/β-catenin signaling cascade (Figure 10). Notably, intervention with CNF effectively abrogated this inhibitory effect, facilitating the intracellular accumulation and activation of β-catenin, which subsequently triggered the transcriptional cascade of downstream effectors. This was evidenced by the robust upregulation of the dermal papilla signature marker Versican (VCAN), the transcription factor (LEF-1), and key cell cycle drivers, including C-Myc and Cyclin D1. The activation of this molecular axis provides a mechanistic basis for how CNF enhances follicular inductivity and bypasses cell cycle arrest, ultimately reconstituting cellular homeostasis against CRF-induced damage.

**Figure 11:**
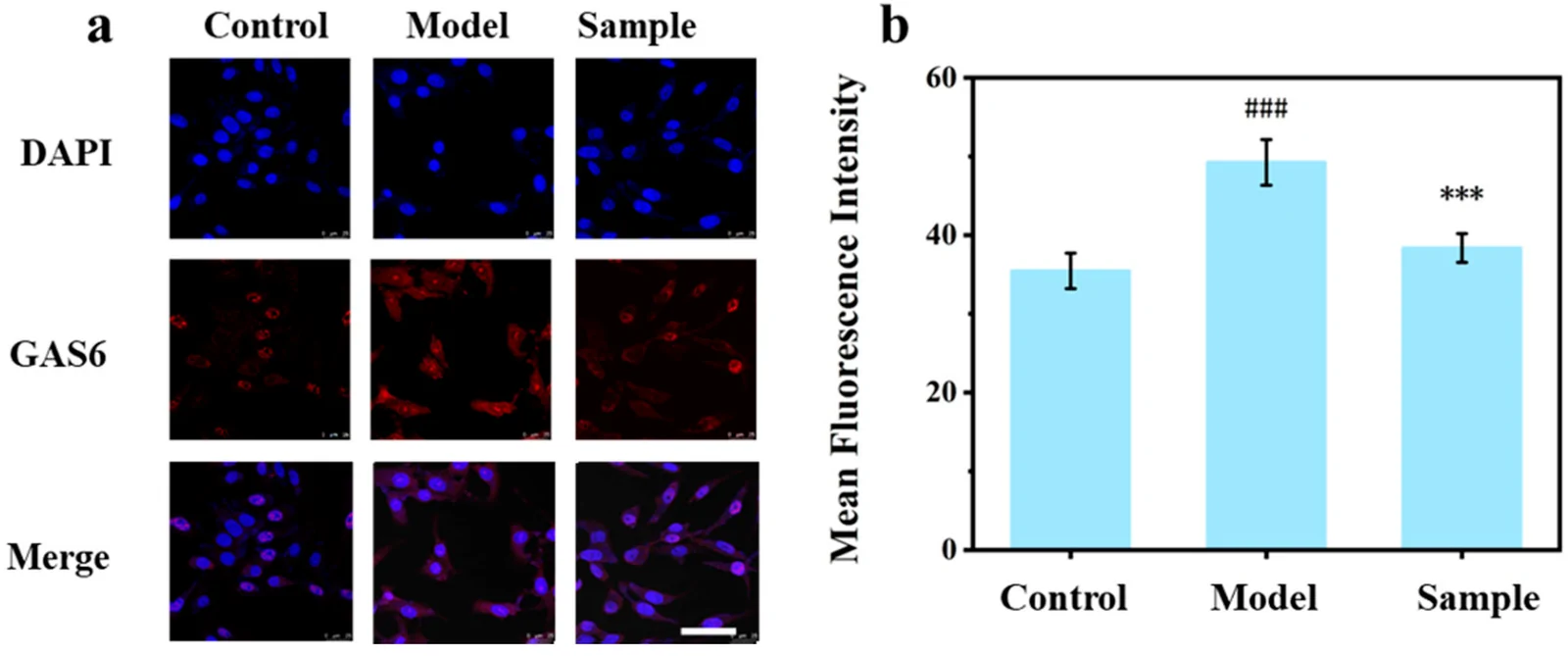
Immunofluorescence staining images of GAS6 under different conditions: Control (untreated), Model (1.0 μM CRF), and CNF (1.0 μM CRF + 50 μg/mL CNF). (**a**) Immunofluorescence image, Scale bars = 50 μm, All images are shown at the same scale bar; (**b**) Corresponding mean fluorescence intensity for GAS6 (### indicated *p* < 0.001 vs. Control group; *** indicated *p* < 0.001 vs. Model group). All data are presented as mean ± SD from three independent biological replicates (*n* = 3).

Furthermore, immunofluorescence analysis revealed that CNF treatment significantly augmented the expression of growth arrest-specific protein 6 (GAS6). As a putative upstream activator of the Wnt/β-catenin cascade and a pivotal pro-survival factor, the restoration of GAS6 levels likely serves as the primordial driver for the CNF-mediated reactivation of proliferative and survival signaling in DPCs (Figure 11) [33,34,35]. These findings delineate a more comprehensive molecular landscape of CNF-mediated antagonism against CRF-induced injury.

### 3.7. CNF Restores the Secretion of Key Hair Growth Factors in CRF-Treated DPCs

**Figure 12:**
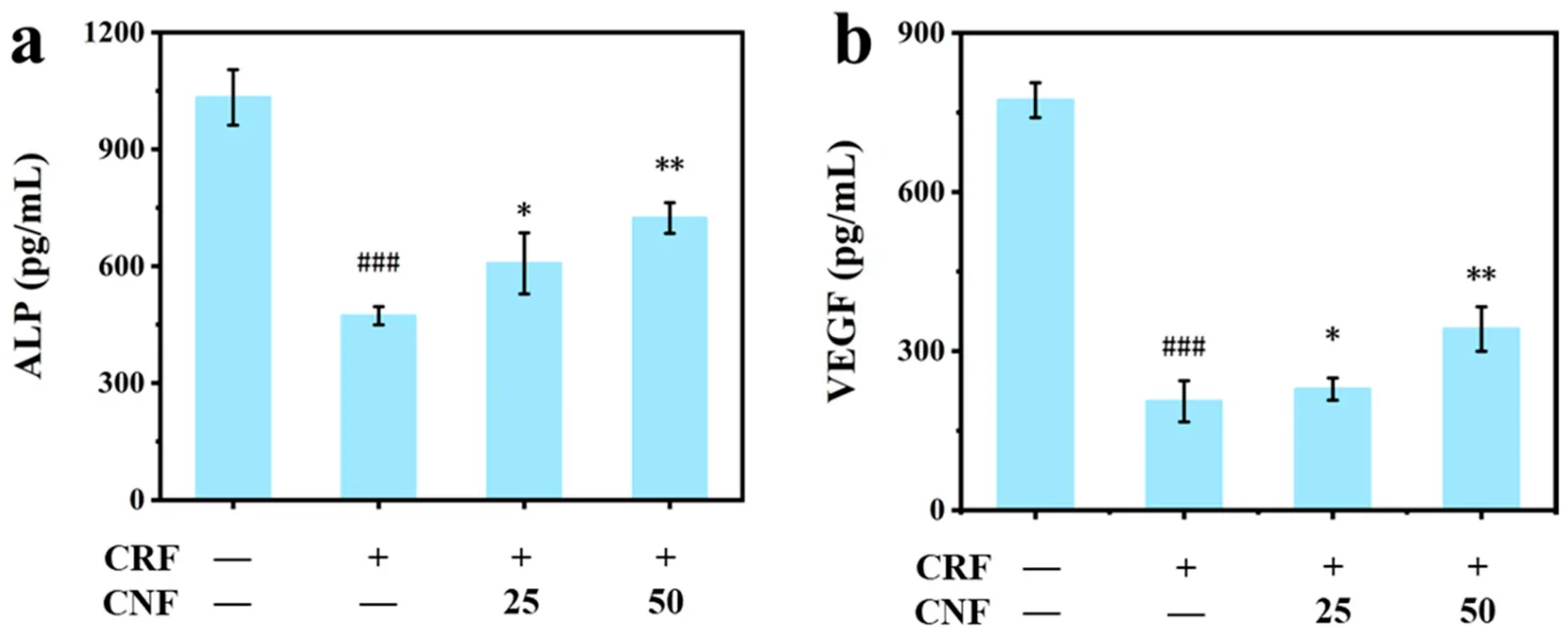
Regulatory effects of CNF on key hair growth factors in DPCs. Cells were treated with 1.0 μM CRF and CNF at concentrations of 0, 25, or 50 μg/mL. (**a**) alp and (**b**) vegfsecretion levels (### indicated *p* < 0.001 vs. control group; * and ** indicated *p* < 0.05 and 0.01 vs. Model group). All data are presented as mean ± SD from three independent biological replicates (*n* = 3).

As shown in Figure 12, CRF treatment significantly suppressed the levels of alp and vegf. Alp is a cardinal marker of DPC hair-inductive potential, while vegf acts as a critical regulator of perifollicular angiogenesis. Intervention with 50 μg/mL CNF significantly restored the secretion of both factors, suggesting that CNF facilitates the reconstruction of the essential hair follicle microenvironment.

**Figure 13:**
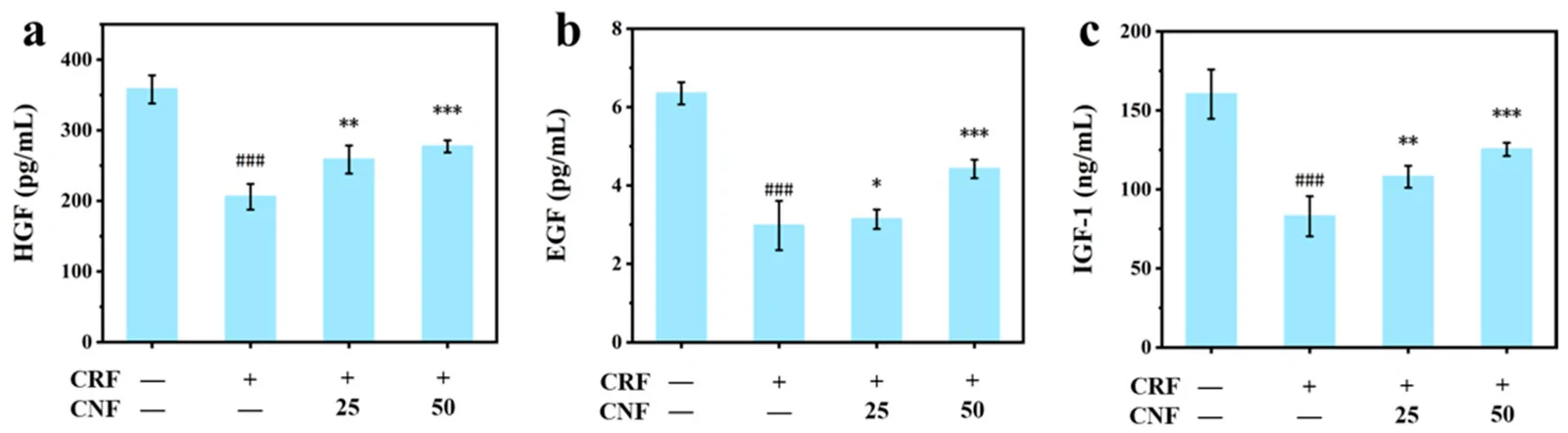
Regulatory effects of CNF on key hair growth factors in DPCs. Cells were treated with 1.0 μM CRF and CNF at concentrations of 0, 25, or 50 μg/mL. (**a**) HGF, (**b**) EGF, and (**c**) IGF-1 secretion levels in the culture medium (### indicated *p* < 0.001 vs. control group; *, **, and *** indicated *p* < 0.05, 0.01, and 0.001 vs. Model group). All data are presented as mean ± SD from three independent biological replicates (*n* = 3).

Furthermore, we assessed the impact of CNF on key paracrine growth factors, including hgf, egf, and igf-1 (Figure 13). Consistent with the observed recovery of hair-inductive markers, CRF-induced deficits in these proliferative factors were effectively reversed by CNF treatment. Collectively, these results indicate that CNF provides more than mere cytoprotection; it actively reconstructs the molecular milieu essential for hair follicle homeostasis and growth, thereby effectively mitigating CRF-induced follicular decline.

## 4. Discussion

Psychological stress has emerged as one of the key extrinsic drivers disrupting hair growth; however, the cellular events linking systemic stress signals to local hair follicle dysfunction remain incompletely elucidated. In this study, by establishing a CRF-induced injury model in DPCs, the hierarchical mechanism by which CNF restores DPC functions by targeting 11β-HSD1, a critical metabolic node [36,37], was assessed.

The most significant finding of this study is the identification of the HPA axis in DPCs as an effective target for intervening in stress-induced alopecia. Furthermore, it is demonstrated that 11β-HSD1 serves as a critical metabolic amplifier driving CRF-induced DPC dysfunction. CNF treatment significantly upregulates the mRNA expression of Wnt5a, β-catenin, LEF-1, and the cell cycle-related genes C-Myc and Cyclin D1. By catalyzing the local conversion of inactive cortisone to active cortisol, 11β-HSD1 amplifies transient stress signals into sustained catabolic signals within the cellular microenvironment of DPCs [38].

Based on this upstream regulatory mechanism, this study systematically elucidates the pleiotropic protective effects of CNF. Compared with previous studies, the present findings provide several distinct contributions. First, while earlier investigations have established the role of 11β-HSD1 in glucocorticoid-mediated DPC dysfunction, our study is the first to demonstrate that a natural plant extract can directly target this enzyme to interrupt the local “stress-cortisol” amplification loop in DPCs. Second, although the TGF-β2/Smad and Wnt/β-catenin pathways have been individually implicated in hair follicle regression and growth, our work reveals that CNF coordinately modulates both pathways through upstream inhibition of 11β-HSD1, thereby achieving simultaneous anti-apoptotic and pro-proliferative effects—a “bidirectional modulation” strategy not previously reported for natural products in the context of stress-induced alopecia. Third, unlike conventional hair growth-promoting agents that primarily act on androgen receptor signaling or growth factor receptors, our study uniquely targets the neuroendocrine metabolic axis (HPA axis-11β-HSD1) within the hair follicle, offering a conceptually novel intervention strategy for stress-related hair loss.

First, CRF-induced upregulation of 11β-HSD1 leads to excessive cortisol accumulation, which in turn triggers a burst of intracellular ROS and suppresses SOD activity, consistent with the literature showing that glucocorticoid overload imposes oxidative burden on DPCs [39]. Second, at the apoptotic level, the protective effect of CNF is mediated by the TGF-β2/Smad signaling axis. CRF significantly activates the TGF-β2/Smad2/3 pathway, whereas CNF effectively antagonizes this signaling cascade and restores the Bcl-2/Bax balance. This active suppression of an established hair follicle regression pathway goes beyond mere cytoprotection, reflecting precise intervention in hair cycle regulation. More importantly, CNF promotes DPC proliferation by restoring Wnt/β-catenin signaling activity [40,41]. CNF treatment significantly upregulates the mRNA expression of Wnt5a, β-catenin, LEF-1, and the cell cycle-related genes C-Myc and Cyclin D1, and also restores the expression of GAS6 [42]. This “bidirectional modulation”—simultaneously inhibiting apoptosis and restoring proliferative capacity—is essential for maintaining hair follicle functional homeostasis. Furthermore, CNF restores the secretion of key paracrine factors [43], such as vegf, alp, hgf, egf, and igf-1 [44,45], indicating that its effects extend from intracellular protection to the reconstruction of the entire hair follicle niche [4,5,7,46].

It is acknowledged that the present study is limited to the in vitro cellular level. Nevertheless, CRF-induced DPC injury serves as a well-established in vitro model for mimicking the microenvironment of stress-related alopecia. This model allows us to dissect the precise molecular mechanisms of CNF under controlled conditions, and the results provide a strong theoretical foundation and clear molecular targets for subsequent in vivo studies. It is planned to further validate the bioactivity of CNF using mouse models of stress-induced alopecia and explore the feasibility of its transdermal administration for human application.

**Figure 14:**
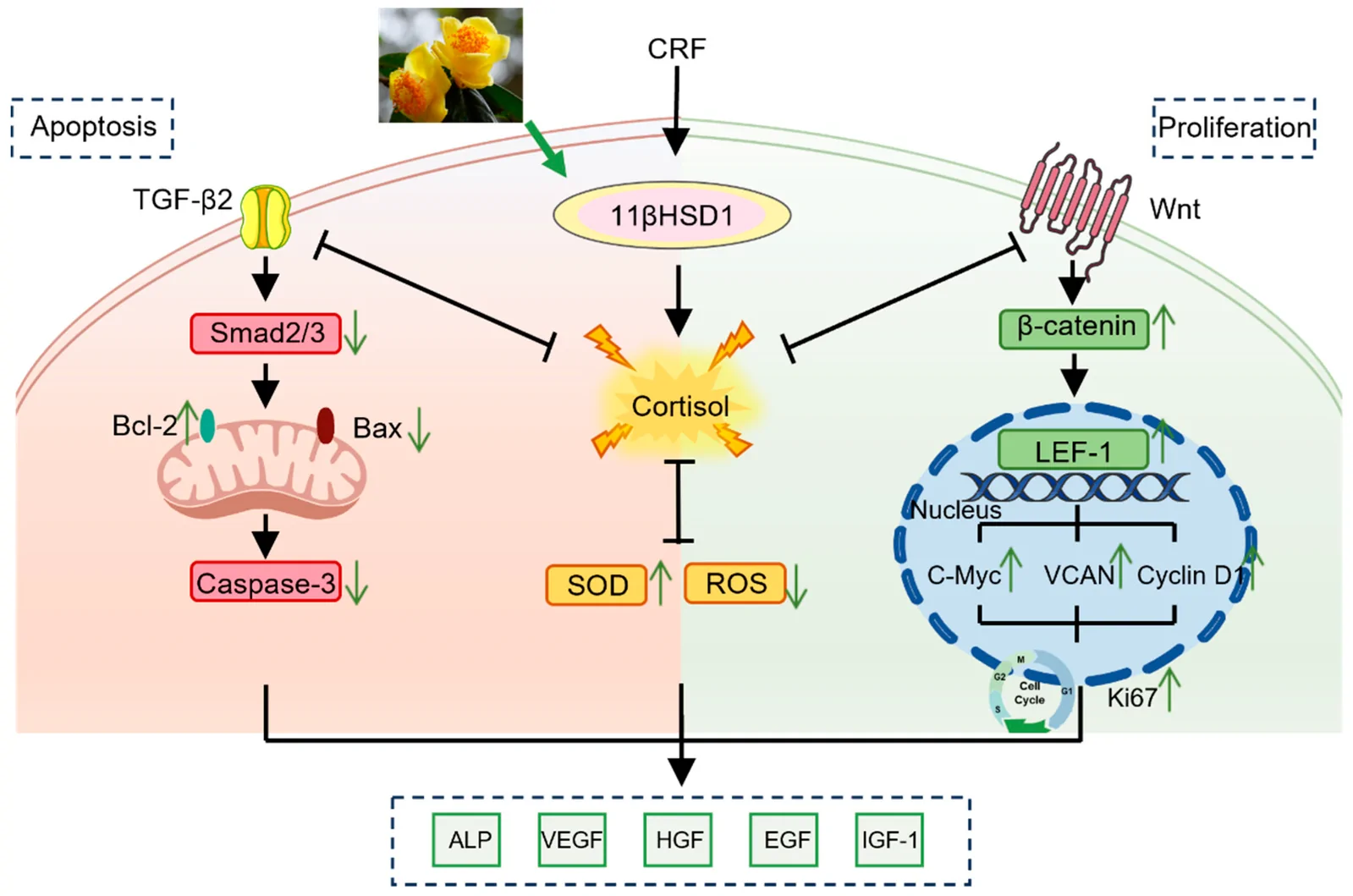
CNF reconstructs the hair growth microenvironment in DPCs through a multi-target regulatory network. Abbreviations: CRF, corticotropin-releasing factor; 11βHSD1, 11β-hydroxysteroid dehydrogenase type 1; TGF-β2, transforming growth factor-beta 2; Smad2/3, Sma- and Mad-related proteins 2 and 3; Bcl-2, B-cell lymphoma 2; Bax, Bcl-2-associated X protein; Caspase-3, cysteine-aspartic protease 3; SOD, superoxide dismutase; ROS, reactive oxygen species; Wnt, wingless-related integration site; β-catenin, beta-catenin; LEF-1, lymphoid enhancer-binding factor 1; C-Myc, cellular myelocytomatosis oncogene; VCAN, versican; Cyclin D1, cyclin D1; Ki67, Ki-67 proliferation marker; ALP, alkaline phosphatase; VEGF, vascular endothelial growth factor; HGF, hepatocyte growth factor; EGF, epidermal growth factor; IGF-1, insulin-like growth factor 1.

In summary, this study demonstrates that CNF, by inhibiting 11β-HSD1, effectively blocks the local amplification loop of the HPA axis, thereby coordinately regulating the TGF-β2/Smad and Wnt/β-catenin signaling pathways and ultimately restoring the hair-inductive phenotype of DPCs (Figure 14). These findings not only provide new insights into the molecular mechanisms underlying stress-induced alopecia but also establish the strong potential of CNF as a natural functional active ingredient for developing interventions against modern psychologically stress-induced hair loss.

## 5. Conclusions

This study demonstrates that CNF exerts its protective effects by specifically targeting the follicle-resident HPA axis, where it inhibits the expression and enzymatic activity of 11β-HSD1, thereby reducing local cortisol overproduction. This intervention effectively interrupts the local “stress–cortisol” amplification loop, thereby preventing the catabolic signaling cascades that drive follicular dysfunction. Furthermore, CNF orchestrates the synergistic modulation of the TGF-β2/Smad and Wnt/β-catenin signaling axes, thereby facilitating the functional reconstruction of DPCs and maintaining the homeostasis of the hair follicle microenvironment. These findings provide novel experimental evidence for elucidating the molecular regulatory mechanisms of stress-induced alopecia and substantiate the potential of 11β-HSD1 as a therapeutic target for natural product intervention. Collectively, CNF represents a promising candidate for development as a natural bioactive ingredient for hair loss prevention. However, as this study was conducted primarily at the in vitro cellular level, further validation using 3D hair follicle models and in vivo systems is warranted to confirm the mechanistic depth and clinical feasibility of these applications.

## Figures and Tables

**Table 1:** Primer design for DPCs.

Primer Name	Primer Sequence
c-Myc	F: 5′-GGTAGTGGAAAACCAGCAGCC-3′
	R: 5′-CTCCTCGTCGCAGTAGAAATACG-3′
Wnt5a	F: 5′-TGCAATGTCTTCCAAGTTCTTCCT-3′
	R: 5′-ATTCATACCTAGCGACCACCAAG-3′
Cyclin D1	F: 5′-AGAGGCGGAGGAGAACAAACAG-3′
	R: 5′-GCGGTAGTAGGACAGGAAGTTGTT-3′
VCAN	F: 5′-TATGGAGATAAGATGGGAAAGGC-3′
	R: 5′-CCGTAATCGCACTGGTCAAA-3′
β-catenin	F: 5′-GCTGAAGGTGCTATCTGTCTGC-3′
	R: 5′-CCTTCCATCCCTTCCTGTTTAG-3′
LEF-1	F: 5′-GCGAATGTCGTTGCTGAGTGTA-3′
	R: 5′-GCTGTCTTTCTTTCCGTGCTAA-3′
TGF-β2	F: 5′-AAAAGCCAGAGTGCCTGAACAAC-3′
	R: 5′-TGCAGCAGGGACAGTGTAAGC-3′
β-actin	F: 5′-CACCCAGCACAATGAAGATCAAGAT-3′
	R: 5′-CCAGTTTTTAAATCCTGAGTCAAGC-3′

**Table 2:** Phytochemical constituents identified in CNF.

Compound	Rt/(min)	Name
1	7.503	Gallic acid
2	18.054	Chlorogenic acid
3	21.369	Caffeic acid
4	22.265	Epigallocatechin gallate
5	27.034	Rutin
6	28.376	Quercetin-7-O-β-D-glucoside
7	29.482	Isoquercitrin
8	31.557	Kaempferol-3-O-rutinoside
9	53.349	Quercetin
10	57.744	Kaempferol

## Data Availability

All data generated or analyzed during this study are included in this published article.

## Abbreviations

The following abbreviations are used in this manuscript:

11β-HSD1	11β-Hydroxysteroid dehydrogenase type 1
ALP	Alkaline phosphatase
CNF	*Camellia nitidissima* flower extract
CRF	Corticotropin-releasing factor
DPCs	Dermal papilla cells
EGF	Epidermal growth factor
GAS6	Growth arrest-specific protein 6
HGF	Hepatocyte growth factor
HPA	Hypothalamic–pituitary–adrenal
IGF-1	Insulin-like growth factor-1
Ki67	Marker of proliferation Ki-67
MTT	3-(4,5-Dimethylthiazol-2-yl)-2,5-diphenyltetrazolium bromide
qRT-PCR	Quantitative real-time reverse transcription polymerase chain reaction
ROS	Reactive oxygen species
SOD	Superoxide dismutase
TGF-β2	Transforming growth factor-β 2
VEGF	Vascular endothelial growth factor
